# Supplementary material for: Doppler imaging detects bacterial infection of living tissue
Source: Commun Biol. 2021 Feb 10;4:178. doi: 10.1038/s42003-020-01550-8 (PMC7876006; doi:10.1038/s42003-020-01550-8)
Supplement: Supplementary file 2 — Supplementary Information [file 42003_2020_1550_MOESM2_ESM.pdf]

Supplementary Information

Doppler Imaging Detects Bacterial Infection of Living Tissue

Honggu Choi[1], Zhe Li[1], Zhen Hua[1], Jessica Zuponcic[2], Eduardo Ximenes[2], John Turek[3], Michael Ladisch[2,4] and David Nolte[1*]

[1]Department of Physics and Astronomy, Purdue University, 525 Northwestern Ave, West Lafayette, IN 47907, USA
[2]Department of Agricultural and Biological Engineering and the Laboratory of Renewable Resources Engineering, Purdue University, West Lafayette, IN 47907, USA
[3]Department of Basic Medical Science, Purdue University, 625 Harrison St, West Lafayette, IN 47907, USA
[4]Weldon School of Biomedical Engineering, Purdue University, West Lafayette, IN 47907, USA

*e-mail: nolte@purdue.edu

Supplementary Note 1. Power Spectral Density and Spectrogram Normalization methods

Three different normalization methods can be used to monitor the shift in spectral content. As an example, the three spectrograms from the identical 10^6 CFU/well *S. enterica* infection spectra are shown in **Supplementary Fig. 1** for three different normalization approaches.

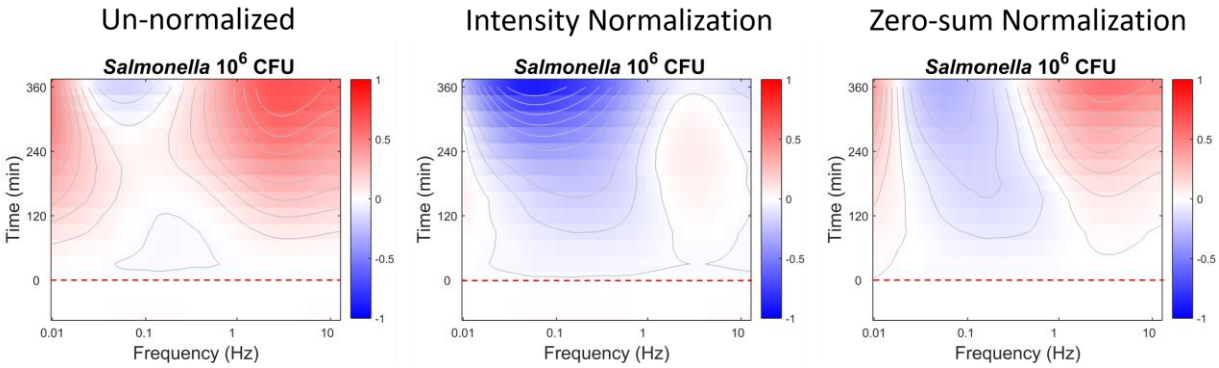

Supplementary Fig. 1. Spectrograms of different normalization methods. Spectrogram of *S. enterica* infection from raw, intensity, and zero-sum normalized power spectral densities after bacterial inoculation (red dashed lines).

Each spectrogram represents a spectrogram obtained by the raw, intensity, and zero-sum normalization, respectively. The un-normalized spectrogram includes changes in sample brightness, and the method is not appropriate for analyzing bacterial infection. The intensity normalization normalizes the power spectral density by the intensity square and is insensitive to changes in the sample brightness. The zero-sum method is only normalized by the power integrated over the limited bandwidth (0.01 Hz ~ 12.5 Hz) which treats the power spectral density as a conserved probability for each time frame. In this example, the un-normalized spectral density increased over time due to the increase of the backscatter brightness. The intensity

normalization shows global suppression across most of the frequency range with a minor spectral enhancement at the high-frequency region. The zero-sum normalization shows a mid-frequency suppression while also showing low and high-frequency enhancements. All of the spectrograms in the paper use intensity normalization because it removes the effects of changing sample brightness while capturing shifts in spectral content.

Supplementary Note 2.1. Infection of 3D Culture with Differing ECM

To demonstrate the different perspectives of the normalization methods, two cohorts of genetically identical host-cells were prepared using two different culture conditions and were subsequently infected by *L. monocytogenes* (10^6 CFU per well). The experiments performed in this paper used a relatively fast growth technique that uses non-adherent Corning U-bottom plates to allow cells to aggregate within two days to form lose spheroid samples. Alternatively, a rotating bioreactor can be used to grow tumor spheroids starting from small clumps of cells. The bioreactor growth requires approximately 3 weeks and produces more tightly bound tumor spheroids with a significantly denser extracellular matrix than for the U-bottom samples.

The signature of *L. monocytogenes* infecting U-bottom spheroids is compared to the infection of bioreactor-grown spheroids in **Supplementary Fig. 2** for the different normalization methods. The lower extracellular matrix (ECM) density of the U-bottom samples produces higher characteristic frequencies than the ECM-rich bioreactor samples. The characteristic frequencies are a factor of 2x to 3x lower for the bioreactor tissue, shown as the dashed lines on the spectrograms in the first column, reflecting the tighter cellular junctions with the ECM than for the U-bottom growth with lower ECM. In the intensity normalization, the infection signature of higher ECM density shows a minor spectral enhancement in the high-frequency region (1~10 Hz) after inoculation while the lower ECM density shows globally suppressed spectral density.

The trend of lower characteristic frequencies with increasing ECM content in the 3D tissue culture has been observed before among different cell lines that have differing adhesion properties [1]. Note that, because the frequency axis spans three orders of magnitude, even a 3x shift in characteristic frequencies still produces spectrograms that are semi-quantitatively similar, as shown in **Supplementary Fig. 2**. When tissue sentinels are used for the assessment of bacterial infection and antibiotic resistance, a standardized cell line and growth technique will be used for all assays, including the negative controls. In this way, the assay captures relative differences among the bacteria or antibiotics, and the underlying characteristic frequency of the tissue sentinels will be identical in all cases.

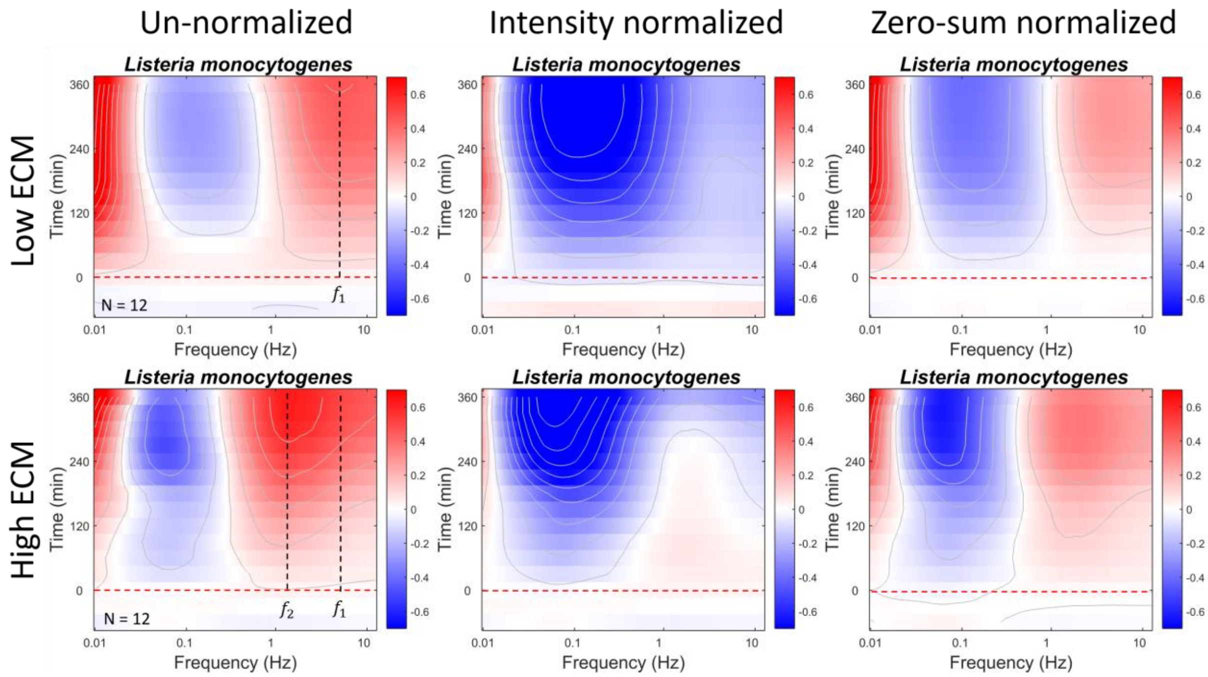

Supplementary Fig. 2. *L. monocytogenes* infection of DLD-1 with different extracellular matrix (ECM) densities. Infection signature by *L. monocytogenes* of DLD-1 with lower ECM density (U-bottom growth) and higher ECM density (bioreactor growth). The red dashed lines represent the *L. monocytogenes* inoculation by 10^6 CFU per well. The average rate of *L. monocytogenes* infection of higher ECM density shows slower development. The vertical dashed lines represent the frequency shift of maximum Doppler spectral density at f_1 to f_2.

Supplementary Note 2.*2. E. coli* growth and pH change in a medium

E. coli growth in growth medium causes the pH to drift because the bacteria consume oxygen and release CO_2. Increasing CO_2 concentration lowers the pH of the medium which perturbs the cellular dynamics of the DLD tissues [2]. The measured pH when 10^6 CFU *E. coli* is inoculated and proliferates inside of the RPMI-1640 medium for 24 hours is shown in **Supplementary Table 1**.

Supplementary Table S1. The pH of the RPMI-1640 growth medium with various agents.

	RPMI-1640 Medium	Medium + E. coli (N = 3)	Medium + E. coli + ampicillin (N = 3)	Medium + E. coli + ciprofloxacin (N = 3)	Medium + DLD-1 (N = 16)
pH	7.4	6.79 ± 0.01	6.76 ± 0.03	8.08 ± 0.01	7.47 ± 0.01

The proliferation of E. coli decreases the pH of the RPMI-1640 medium. *E. coli* treated with ampicillin decreased pH slightly more than without ampicillin which may be related to the *E. coli* ampicillin resistance mechanism. β-lactamase molecules secreted by ampicillin-resistant *E. coli* neutralize ampicillin molecules and generates additional CO_2 during the process [3, 4]. *E. coli* treated with ciprofloxacin shows a complete suppression of *E. coli* proliferation.

Supplementary Note 3. Spectral Bands

The time traces of selected spectral ranges are shown in **Supplementary Fig. 3** for the rheology band (10 mHz) and the organelle transport band (2 Hz – 6Hz) for an applied exposure of 10^7 CFU per well [5]. The rheology band is associated with slow cellular shape changes and also with reduced speeds of mid-frequency processes related to membranes or larger organelles like the nucleus. At the high bacterial load of 10^7 CFU per well there is a strong non-monotonic time dependence of the spectral density of the rheology band as a maximum appears after approximately 2 hours and then the signal decreases. In the case of *S. enterica* and *L. monocytogenes,* the secondary minimum gives way to a later increase, while for the non-pathogenic *E. coli* and *L. innocua* the secondary minimum does not occur. The organelle band presents a relatively "clean" signal because the only intracellular constituents that contribute to this spectral range are the smaller organelles and vesicles. The spectral density of the organelle band is enhanced for all but *E. coli* infection. Organelle and vesicle transport are associated with active cellular responses to xenobiotics as well as with early-stage apoptosis. The higher frequencies may also be associated with the motion of the bacteria themselves.

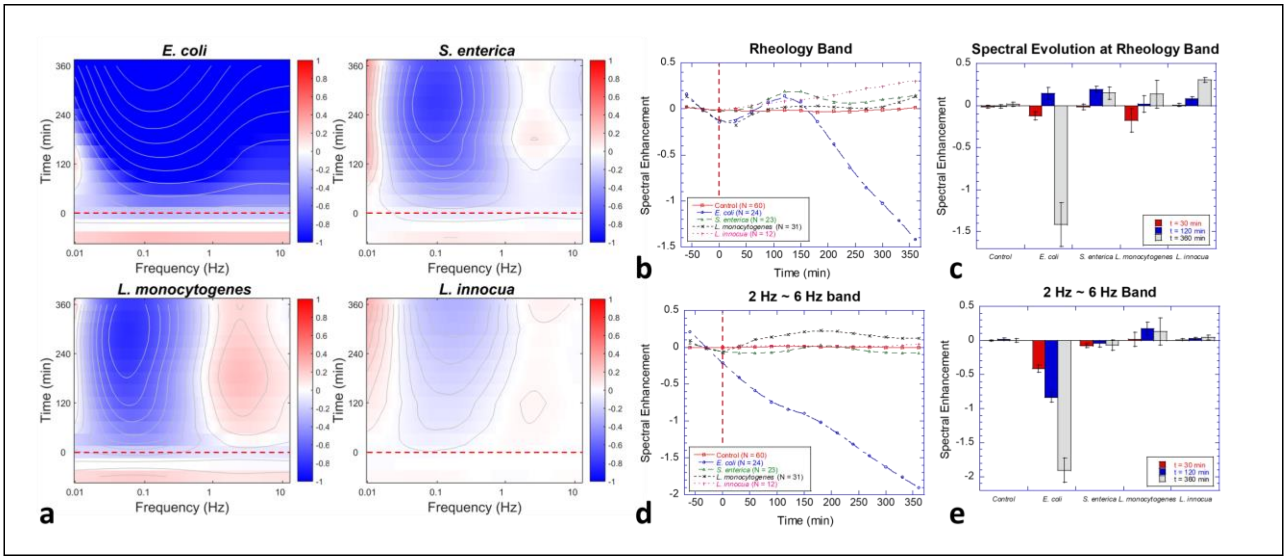

Supplementary Fig. 3. Time development of the relative spectral changes for a dose of 10^7 CFU per well. **a**, The bacteria are added at 0 minutes followed by rapid changes in the spectral power (intensity normalized). **b**, The low-frequency limit for the rheology band. All bacterial strains showed temporal variation in this band. *E. coli* infection displays the strongest suppression. The two pathogenic strains, *S. enterica,* and *L. monocytogenes* show non-monotonic increases, with a decrease after the first maximum, followed by a long-term increase. Long-term increases for the rheology band have been associated with blebbing or the formation of apoptotic bodies associated with either uncontrolled or controlled cell death. *L. innocua* show a slow and monotonic increase. **c**, Change in the Doppler rheology band for three selected times and standard errors. **d**, Time dependence of the high-frequency band associated with organelle transport. *L. monocytogenes* show strong initial increases within one hour followed by a slow decrease at longer times. *S. enterica* show a weak enhancement at 3 hours after inoculation. The case for *E. coli* shows strong suppression consistent with an overall inhibition of Doppler activity. **e**, Change in the Doppler organelle transport band at three selected times.

Supplementary Note 4. Backscattering brightness and NSD for 10^6 CFU inoculation

Tissue sentinels were exposed to different initial numbers of bacteria. In the main text, Fig. 2 shows the OCI, MCI, NSD, and BSB for exposure to 10^7 CFU. **Supplementary Fig. 4** is a comparison figure for exposure of 10^6 CFU. The responses to the initial bacterial dose are similar in both figures indicating that the infection signature is not limited by the initial bacteria exposure at least above 10^6 CFU. Therefore, the infection response is transport-limited at these exposure levels.

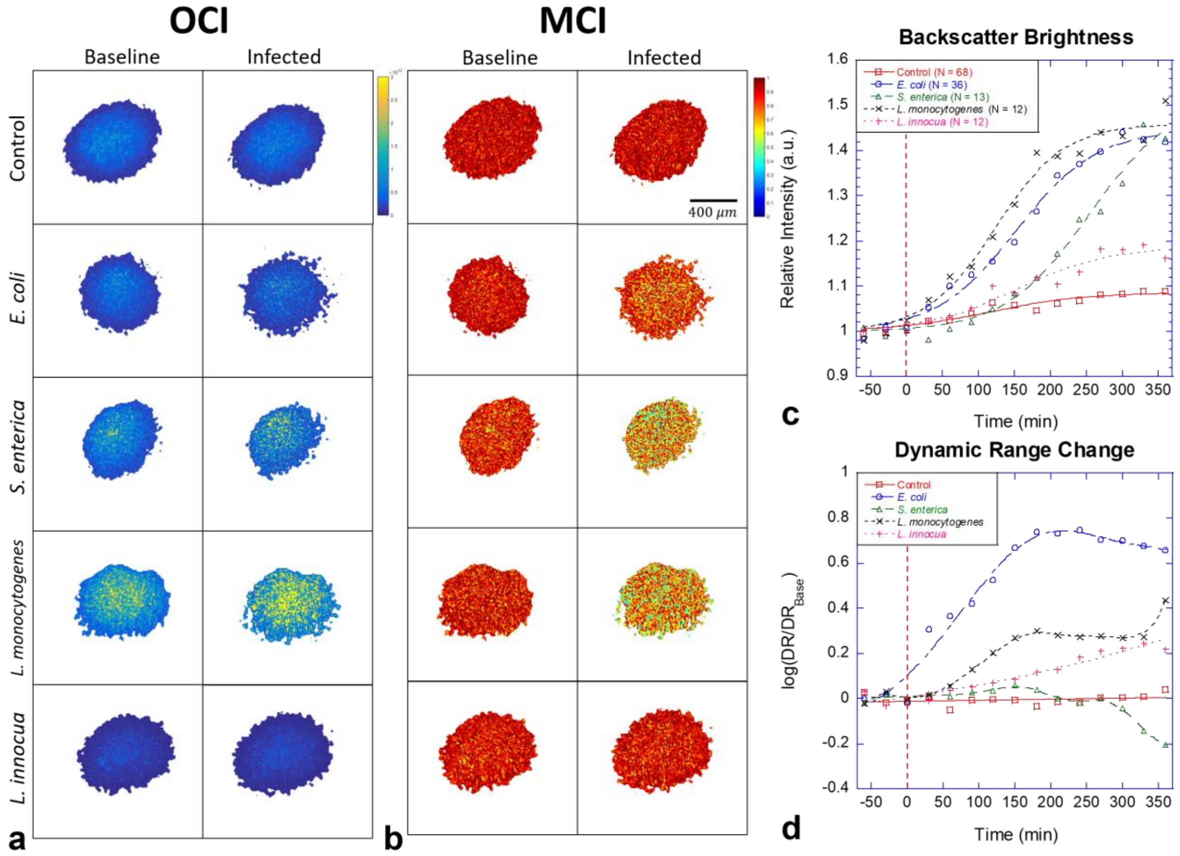

Supplementary Fig. 4. Inoculation with 10^6 CFU. a, Optical coherence images (OCI) and **b**, corresponding motility contrast images. The responses are shown in **c**, backscattering brightness, and **d**, Dynamic range change of spectral density. Compare this figure (at 10^6 CFU) to Fig. 2 (at 10^7 CFU). (*L. innocua* data are quoted from Fig. 2)

Supplementary Note 5. Levy Alpha spectroscopy of Suppressed DLD-1 metabolism by Chemical Agents

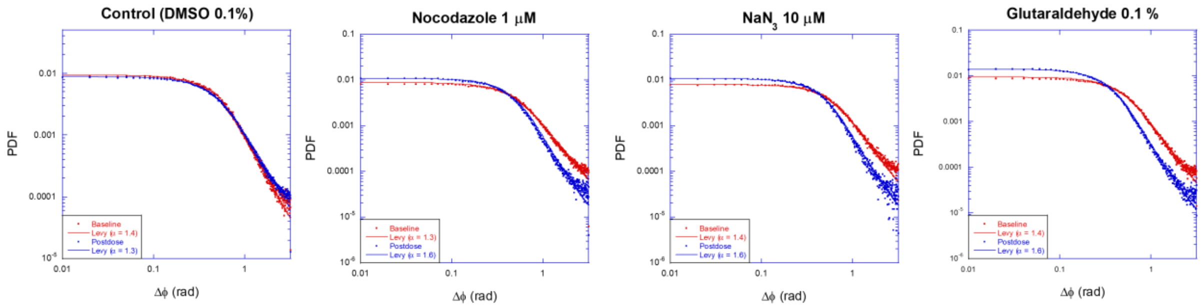

Supplementary Fig. 5. Examples of Levy-Alpha spectroscopy of DLD-1. DLD-1 spheroids were treated by chemical agents such as nocodazole, sodium azide, and glutaraldehyde. All the chemical agents are expected to hinder cellular activities and reduce transport (less ballistic). The baselines were measured and all had fitted alpha values between 1.3 and 1.4. After 24 hours under the respective treatments, the postdose PDFs were measured, and the alpha values were fitted. The statistical results are shown in **Supplementary Table 2**. The inhibitory drugs increased the Lévy alpha by nearly 0.2 in all cases.

Supplementary Table 2. The averages and standard deviations of DLD-1 responses. The Levy-Alpha of control remained almost the same after 24 hours. However, other groups treated by chemical agents suppressing DLD-1 metabolism resulted an increase in the Levy-Alpha values (less ballistic). The replicate numbers of Levy-Alpha values are $N_{control}$ = 8, $N_{Nocodazole}$ = 8, N_{NaN3} = 7, and $N_{Glutaraldehyde}$ = 5.

Group	Control (DMSO 0.1%)	Nocodazole (1 μM)	NaN_3 (10 μM)	Glutaraldehyde 0.1 %
$\Delta\alpha$	-0.06 ± 0.16	0.18 ± 0.04	0.19 ± 0.07	0.18 ± 0.12

Supplementary Note 6. Key Metrics for Biodynamic Imaging

There are approximately 5 spectral bands that can be defined in the drug-response spectrograms. These are defined in **Supplementary Table 3** with their presumed biophysical origins.

Supplementary Table 3. Spectral Bands

Band Name	Frequency Range	Speed Range	Biophysics Origins
Cell Motility Band	10 mHz	3 nm/s	Crawling
Rheology Band	12.5 mHz – 100 mHz	4 nm/s – 30 nm/s	Shape change
Mid Band	100 mHz – 1 Hz	30 nm/s – 300 nm/s	Membrane/Nuclear
High Band	1 Hz – 10 Hz	300 nm/s – 3 μm/s	Organelle transport
Nyquist Band	12.5 Hz	4 μm/s	Vesicle transport

The time-frequency spectrograms are converted into feature vectors with elements associated with parts or patterns of the spectrograms. In addition to spectrogram-based features, there are also preconditions (like sample brightness and dynamic range, etc.) as well as drug-induced changes in these preconditions. All the raw biomarkers are defined in **Supplementary Table 4**. The time-frequency decomposition is approached both globally and locally. Global patterns are generated as low-order Legendre polynomials. These polynomials are taken as an inner product over the spectrograms to generate Legendre coefficients that represent the global features of the spectrograms. Only orders 0, 1, and 2 are used along the frequency and time axes to generate 9 global features. Local patterns are simply low, mid, and high-frequency bands with average, linear, and quadratic time dependence for 9 local features. The preconditions consist of normalized standard deviation (NSD), backscatter brightness (BSB), number of pixels in the sample mask (NCNT), the spectral dynamic range (DR), the Nyquist floor (NY), the knee frequency (KNEE), the half-width (HW), the spectral slope (S) and the linear slope (SF). (Note that NCNT, KNEE, and S are subject to non-convex regression errors and may be down-selected as features.) Each precondition is changed by the drug treatment, providing additional features that are the changes in the preconditions from baseline to endpoint of the assay. There are 27 drug-response features: 18 are based on spectrograms and 9 are drug-induced changes in preconditions. These 27 features are concatenated for each drug to create a feature vector of 27*Ndrug elements.

Supplementary Table 4. Definitions of Biodynamic Biomarkers

	Biomarker Name	Description
		**Global Spectral Biomarkers**
1	ALLF0	All frequencies. All times
2	SDIP0	Blueshift: All times
3	CDIP0	Middle-out: All times
4	ALLF1	All frequencies. Linear time dependence
5	SDIP1	Blueshift: Linear time dependence
6	CDIP1	Middle-out: Linear time dependence
7	ALLF2	All frequencies. Quadratic time dependence
8	SDIP2	Blueshift: Quadratic time dependence
9	CDIP2	Middle-out: Quadratic time dependence
		**Local Spectral Biomarkers**
10	LOF0	Low-frequencies: All times
11	MID0	Mid-frequencies: All times

12	HI0	Hi-frequencies: All times
13	LOF1	Low-frequencies: Linear time dependence
14	MID1	Mid-frequencies: Linear time dependence
15	HI1	Hi-frequencies: Linear time dependence
16	LOF2	Low-frequencies: Quadratic time dependence
17	MID2	Mid-frequencies: Quadratic time dependence
18	HI2	Hi-frequencies: Quadratic time dependence
		**Change in Precondition**
19	DNSD	Change in normalized standard deviation (NSD)
20	DBSB	Change in back-scatter brightness (BSB)
21	DNCNT	Change in number of pixels (NCNT)
22	DDR	Change in dynamic range (DR)
23	DNY	Change in Nyquist floor (NY)
24	DKNEE	Change in knee frequency (KNEE)
25	DHW	Change in half-width (HW)
26	DS	Change in Slope (S)
27	DSF	Change in linear slope (SF)
		**Precondition**
28	NSD	Normalized standard deviation (NSD)
29	BSB	Back-scatter brightness (BSB)
30	NCNT	Number of pixels (NCNT)
31	DR	Dynamic range (DR)
32	NY	Nyquist floor (NY)
33	KNEE	Knee frequency (KNEE)
34	HW	Half-width (HW)
35	S	Slope (S)
36	SF	Linear slope (SF)
37	B0	Baseline: all frequencies
38	B1	Baseline: linear frequency
38	B2	Baseline: quadratic frequency
40	DQ	Data Quality

Many drugs produce common spectrogram patterns. These are defined and described in **SupplmentaryTable 5** along with presumed biophysical mechanisms.

Supplementary Table 5. Common Global Spectrogram Patterns

**Pattern Name**	**Pattern**	**Char. Biomarker**	**Biophys. Origins**
Suppression	Blue	ALLF (neg)	Overall suppression of motion
Enhancement	Red	ALLF (pos)	Overall enhanced motion
Red Shift	Red - Blue	SDIP (neg)	Average speeds decrease
Blue Shift	Blue-Red	SDIP (pos)	Average speeds increase
Middle-Out	Red-Blue-Red	CDIP (pos)	Apoptosis/interrupted transport
Middle-In	Blue-Red-Blue	CDIP (neg)	More persistent transport
Drift Red	Time drift left	SDIP1 (pos)	Time drift to lower speeds
Drift Blue	Time drift right	SDIP1 (neg)	Time drift to higher speeds
Skew Red	Transition	CDIP1 and SDIP1(-)	Time change in pattern: rd-shift
Skew Blue	Transition	CDIP1 and SDIP1(+)	Time change in pattern: bl-shift

References

1. Merrill, D., et al., *Digital holography of intracellular dynamics to probe tissue physiology.* Applied Optics, 2015. **54**(1): p. A89-A97.
2. Nolte, D.D., et al., *Holographic tissue dynamics spectroscopy.* Journal of Biomedical Optics, 2011. **16**(8).
3. Fratamico, P.M., et al., *Construction and Characterization of Escherichia coli 0157:H7 Strains Expressing Firefly Luciferase and Green Fluorescent Protein and Their Use in Survival Studies.* Journal of Food Protection, 1997. **60**(10).
4. Fernandesa, R., P. Amadord, and C. Prudencio, *b-Lactams: chemical structure, mode of action and mechanisms of resistance.* Reviews in Medical Microbiology, 2013. **24**(1).
5. Li, Z., et al., *Doppler fluctuation spectroscopy of intracellular dynamics in living tissue.* Journal of the Optical Society of America a-Optics Image Science and Vision, 2019. **36**(4): p. 665-677.
