# Supplementary material for: Doppler imaging detects bacterial infection of living tissue
Source: Commun Biol. 2021 Feb 10;4:178. doi: 10.1038/s42003-020-01550-8 (PMC7876006; doi:10.1038/s42003-020-01550-8)
Supplement: Supplementary file 3 — Description of Additional Supplementary Files [file 42003_2020_1550_MOESM3_ESM.pdf]

Description of Additional Supplementary Files

File Name: Supplementary Data 1

Description: Source data of the graphs in the main figures

Supplementary Data 1: Source data of figure 2c and d. The unit of the backscatter brightness is normalized by the average backscatter brightness of baseline measurements.

Supplementary Data 2: Source data of figure 3a. The units of the x-axis is the frequency (Hz) and the y-axis is the normalized spectral density (a.u.).

Supplementary Data 3: Source data of figure 3b. The figures are plotted in Frequency axis (Hz) and Time axis (min).

Supplementary Data 4: Source data of figure 4b. The pixel size is 8 μm.

Supplementary Data 5: Source data of figure 4c.

Supplementary Data 6: Source data of figure 5e, f, and g

Supplementary Data 7: Source data of figure 6. The figures are plotted in Frequency axis (Hz) and Time axis (min).

File Name: Supplementary Data 2 (Available at: https://figshare.com/articles/dataset/fig2ab_mat/13235075)

Description: Source data of OCI and MCI demonstrated in Fig. 2a and b. One pixel represents 8 μm distance.

Variables:

OCI_control_baseline: OCI image of control before applying medium

OCI_control_infected: OCI image of control after applying medium

OCI_E_coli_baseline: OCI image of control before *E. coli* inoculation

OCI_ E_coli _infected: OCI image of control after *E. coli* inoculation

OCI_L_innocua_baseline: OCI image of control before *L. innocua* inoculation

OCI_ L_innocua _infected: OCI image of control after *L. innocua* inoculation

OCI_L_monocytogenes_baseline: OCI image of control before *L. monocytogenes* inoculation

OCI_ L_monocytogenes _infected: OCI image of control after *L. monocytogenes* inoculation

MCI_S_enterica_baseline: MCI image of control before *S. enterica* inoculation

MCI_ S_enterica _infected: MCI image of control after *S. enterica* inoculation

MCI_control_baseline: MCI image of control before applying medium

MCI_control_infected: MCI image of control after applying medium

MCI_E_coli_baseline: MCI image of control before *E. coli* inoculation

MCI_ E_coli _infected: MCI image of control after *E. coli* inoculation

MCI_L_innocua_baseline: MCI image of control before *L. innocua* inoculation

MCI_ L_innocua _infected: MCI image of control after *L. innocua* inoculation

MCI_L_monocytogenes_baseline: MCI image of control before *L. monocytogenes* inoculation

MCI_ L_monocytogenes _infected: MCI image of control after *L. monocytogenes* inoculation

MCI_S_enterica_baseline: MCI image of control before *S. enterica* inoculation

MCI_ S_enterica _infected: MCI image of control after *S. enterica* inoculation
